# The Ontogeny of Vocal Rhythms in a Non‐Human Primate

**DOI:** 10.1111/desc.70189

**Published:** 2026-04-23

**Authors:** Lia Laffi, Teresa Raimondi, Chiara De Gregorio, Daria Valente, Walter Cristiano, Filippo Carugati, Valeria Ferrario, Valeria Torti, Jonah Ratsimbazafy, Cristina Giacoma, Andrea Ravignani, Marco Gamba

**Affiliations:** ^1^ Department of Life Sciences and Systems Biology University of Turin Turin Italy; ^2^ Department of Human Neurosciences Sapienza University of Rome Rome Italy; ^3^ Fondazione ZOOM Cumiana Italy; ^4^ Unit of EcoHealth, Department of Environment and Health Italian National Institute of Health Rome Italy; ^5^ Groupe dʹÉtude et de Recherche sur les Primates de Madagascar (GERP) Fort Duchesne Antananarivo Madagascar; ^6^ Comparative Bioacoustics Group Max Planck Institute for Psycholinguistics Nijmegen The Netherlands; ^7^ Center for Music in the Brain, Department of Clinical Medicine Aarhus University & The Royal Academy of Music Aarhus Denmark; ^8^ Research Center of Neuroscience ‘‘CRiN‐Daniel Bovet’’ Sapienza University of Rome Rome Italy; ^9^ Institute of Cognitive Sciences and Technologies National Research Council Rome Italy

**Keywords:** development, indri, musicality, singing primate, rhythm

## Abstract

Rhythm is a fundamental aspect of human behaviour, and musical rhythm provides one of its most elaborate instances. Unlike speech, this rhythmic behaviour is characterized by the production of temporal patterns structured around small‐integer ratios, which emerge early in life and change systematically across development. Whether such developmental trajectories are uniquely human or reflect broader biological constraints remains an open question. Here, we adopt a comparative developmental approach to map the ontogeny of rhythmic structure in the vocalizations of a non‐human primate, the singing lemur *Indri indri*. We recorded songs from individuals of different age classes and quantified temporal organization by measuring inter‐onset intervals between successive note onsets. From these intervals, we computed rhythmic ratios between adjacent units and assessed their correspondence to small‐integer values. We find that isochrony (1:1 ratios), a core feature of human rhythm, is present from the earliest stages of vocal production. Over development, indris produce an increasing diversity of rhythmic structures corresponding to simple numerical relationships between adjacent intervals. This similarity to humans contrasts with three key differences. First, in indri, binary ratios (1:2 and 2:1) emerge gradually. Second, rhythmic precision around small‐integer ratios does not systematically increase with age. Third, developmental trajectories differ between males and females. Together, these findings reveal both shared and divergent developmental pathways of rhythm production in humans and non‐human primates, suggesting that early‐emerging temporal regularity (i.e., isochrony) may reflect conserved biological constraints, whereas later‐developing aspects of rhythmic structure are shaped by species‐specific developmental processes.

## Summary

- A non‐human primate produces temporally regular vocal rhythms from the earliest stages of vocal development
- Isochrony (1:1 timing), a core feature of human musical rhythm, is present across sexes and all developmental stages
- Similarly to humans, rhythmic complexity increases over development through the emergence of additional small‐integer ratios between adjacent intervals
- Unlike humans, the precision of rhythmic regularity does not increase with age and follows sex‐specific developmental trajectories

## 1 Introduction

### 1.1 The Nature of Musical Rhythm: Developmental and Cross‐Cultural Approaches

Among the many existing definitions of rhythm, one captures a generalized commonality: rhythm denotes the structuring of events in time (Toussaint 2019). Rhythmic capacities denote the biological, psychological and neural underpinnings needed to produce and perceive rhythm. In humans, focusing on the sensory‐motor system, some rhythmic capacities are present at birth or very early in life. These include rhythm production, in terms of spontaneous stereotypical motor (Laudanska et al. 2022; Rocha et al. 2021a; Thelen 1979), respiratory (De Vries and Fong 2006), sucking (Wolff 1968) and vocal (babbling, Ejiri 1998; cries, Wermke and Mende 2009) rhythms. Vocal and motor output are temporally intertwined (Provasi et al. 2014), and the precision of their synchronization increases until 24‐months of age (Borjon et al. 2024). Conversely, children's acquisition of some adult‐like capacities underlying musical rhythmic perception and production develops over time, through training and cultural interactions (Hannon et al. 2018). For instance, with age, spontaneous infant drumming becomes more rhythmically regular (Rocha et al. 2021a) and tempo matching improves and expands to a wider tempo range with age (Provasi and Bobin‐Bègue 2003; Rocha et al. 2021). The development of rhythmic abilities continues into adulthood. Children begin to demonstrate the ability to synchronize with a rhythmic sequence around the age of 10 (Drake et al. 2000), and rhythmic entrainment continues to develop throughout adolescence (Thompson et al. 2015). Moreover, adult‐like beat perception does not fully mature until adolescence (Nave et al. 2023). In brief, human rhythmic capacities showcase a wonderful mosaic of fairly automatic processes already active at birth, combined with abilities acquired over the lifespan and culturally‐nuanced traits. There are three complementary approaches to disentangle these factors: developmental, cross‐cultural and comparative.

Obviously, the aforementioned developmental approach provides a key contribution to pinpointing the nature of rhythmic abilities. Complementarily, cross‐cultural work in adults addresses two related questions. First, the cross‐cultural experimental testing provides a direct comparison of what happens when similar organisms are raised in different musical cultures; with some exceptions, however, this testing is costly and can only target a subset of abilities (Jacoby et al. 2024). Alternatively, corpus studies (Mehr et al. 2019) can pinpoint similarities and differences between rhythm, intended as the cultural artifact generated by rhythmic capacities (Savage et al. 2015). If a specific rhythm feature is found in all music cultures then it is likely a direct, downstream product of specific rhythmic capacities. Alternatively, if some traits are only found in a few cultures, there may be a more tenuous correspondence between rhythm and rhythmicity.

### 1.2 Rhythmic Categories: Universality and Music‐Specificity

The corpus approach has delivered a precise map of the very few ‘musical rhythm universals’ across human cultures. One of these is the production of rhythmic categories that coincide with small‐integers (Roeske et al. 2020; Savage et al. 2015). These occur when the time intervals between adjacent note onsets (i.e., inter‐onset intervals) are integer multiples of each other (Jacoby et al. 2024; Jacoby and McDermott 2017; Savage et al. 2015). When calculating the *ratio* between two adjacent inter‐onset intervals, a random process in time generates a ratio distribution; this is the null hypothesis of lack of rhythmicity (De Gregorio et al. 2021b; Jadoul et al. 2025a, 2025). Conversely, rhythmic organisation occurs when ratio distributions show clusters of high frequency of occurrence around specific values; these are called *rhythmic categories* (Roeske et al. 2020). The simplest rhythm category derives from an isochronous signal: when adjacent inter‐onset intervals have the same duration, a constant 1:1 rhythmic ratio ensues. Other rhythmic categories include 1:2, capturing a sequence of intervals where an interval typically lasts double than its preceding one.

In humans, a crucial feature of rhythmic categories is their relatively high abundance in music compared to speech (Vanden Bosch Der Nederlanden et al. 2023). While in music temporal intervals are quantized multiples of each other, the same is rarely true for speech (but see Cummins and Port 1998). These categories are quantified and compared via multiple datasets (Clayton et al. 2019, Clayton et al. 2022; Jadoul et al. 2025b; Roeske et al. 2020). The term ‘rhythmic categories’ is also used in experimental psychology to refer to cognitive priors, in other words the putative rhythmic ability underlying the rhythmic structure measured in corpora. These priors have been measured via iterated reproduction tasks, where participants are asked to imitate a rhythmic sequence, and the deviations between original and copy are used to reconstruct these cognitive priors (e.g., Jacoby et al. 2024; Jacoby and McDermott 2017; Ravignani et al. 2016). A recent study investigated the developmental trajectory of these rhythmic biases, showing adult‐like biases towards 2:1 and 1:2 in children as young as 6‐years‐old but also that more complex ratios including 3:2 emerged with age in a culture‐specific fashion (Nave et al. 2024). How developmentally constrained are these rhythmic categories? Which features are due to fairly automatic processes already active at birth, which ones to abilities acquired over the lifespan and which are culturally‐modulated traits? Without comparative developmental data, it remains unclear which aspects of rhythmic development reflect human‐specific learning and which reflect more general mammalian developmental constraints.

### 1.3 The Comparative Approach and Our Animal Model: A Singing Primate

**FIGURE 1:**
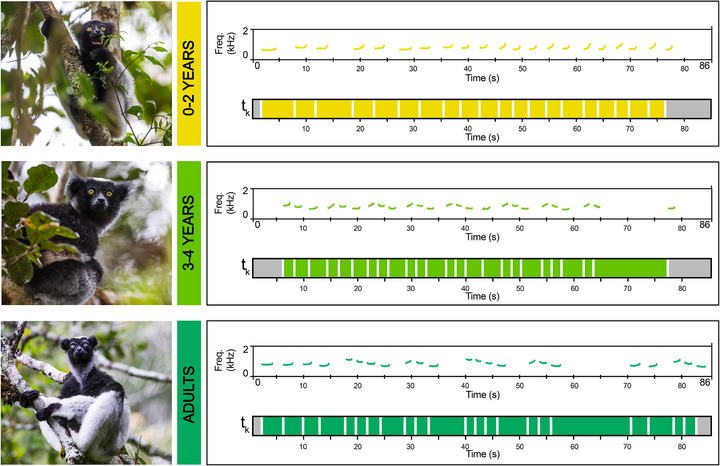
On the left, pictures of individuals of *Indri indri* at the three age classes (0–2 years, 3–4 years, adult; pictures copyright of *Filippo Carugati*). On the right, exemplary spectrograms and inter‐onset intervals (*t_k_*) of individual contributions at different age classes. The spectrograms show the fundamental frequency of three female individual contributions. The *t_k_* are highlighted for the entire duration of the contribution as coloured bars, separated by white lines, which correspond to the onsets of every emitted unit.

Until 2020, the vocal production of rhythmic categories was thought uniquely human. However, in recent years, the quantification of rhythmic ratios in a growing number of spontaneous animal vocalizations (e.g., mammals: De Gregorio et al. 2025a, De Gregorio et al. 2025b; Demartsev et al. 2022; Raimondi et al. 2023; e.g., birds: Roeske et al. 2020; Xing et al. 2022) suggested that, in some cases, the dominant temporal architecture corresponds to rhythmic categories falling around small‐integer ratios, similarly to music. Importantly, the mere observation of these rhythms in animal vocal output does not entail any perceptual categorical process in the observed species. Nonetheless, it can help reconstruct the evolutionary prerequisites, in terms of mechanisms and functions, for vocal rhythm production in mammals, a necessary step toward understanding the evolution of rhythmic capacities in humans. Mammalian models to study the ontogeny of rhythm production, and in particular rhythmic categories, are rare. Numerous authors have suggested that non‐human primate songs provide an opportunity to map the evolution of human musicality, that is, the biologically‐rooted set of capacities and proclivities that allows us to generate and enjoy music (Fitch 2015; Geissman 2000). Primate songs are complex vocalisations characterized by a sequence of frequency‐modulated units, arranged in phrases (De Gregorio et al. 2022). Songs contain units separated by silent gaps: the timespan of these two durations (unit + silent gap) together can vary and constitutes an inter‐onset interval. Very similar to human music, one main unit of temporal organization in primate songs is the inter‐onset interval (e.g., Gamba et al. 2016; Raimondi et al. 2023; Roeske et al. 2020). The singing lemur *Indri indri* (Figure 1) is one of the few mammals showing not only calling, but also singing behaviour (De Gregorio et al. 2022). Indri song supports communication within and between groups, maintenance of a territory's occupation and defence of borders during group encounters (Bonadonna et al. 2020; Torti et al. 2013). One particular display, the advertisement songs, serves crucial functions in this species (Torti et al. 2013). It mediates the formation of new groups, advertises resource‐holding potential, and reduces the probability of encounters by regulating group movements in the forest and by avoiding physical fights (Pollock 1986). Indris live in small groups of two to six individuals: a reproductive pair and their offspring (Bonadonna et al. 2019). Females reach sexual maturity earlier than males (De Gregorio et al. 2021a) and start to sing around 12–16 months of age (females at 11.88 months, males at 14.76; Brunod et al. 2025; De Gregorio et al. 2021a). The estimated lifespan is up to 30 years for male indris and up to 35 years for females (Rolle et al. 2021).

Adult indris are, to date, the singing primates producing the highest number of shared rhythmic categories with human music (De Gregorio et al. 2021b; De Gregorio et al. 2024a). The presence of the three small‐integer ratios detected in the adult indris’ song has been linked to the song's syntactic structure (De Gregorio et al. 2021b; De Gregorio et al. 2024a). The song consists of units organised into phrases, resulting in the alternation of short (within a phrase) and long (between phrases) intervals (Figure 1). For instance, short intervals within a phrase will produce a local isochronous pattern (1:1 ratio), while long intervals between phrases, with double the duration of short intervals, will produce the other two ratios (1:2 and 2:1 ratios; De Gregorio et al. 2021b; De Gregorio et al. 2024a).

Indris hence constitute to date the only singing primate model showing three rhythmic categories in singing behaviour, similar to human music. Past comparative work has provided a positive answer to the question: Can non‐human primates produce some of the rhythmic categories we see in human music? Singing in young indris undergoes substantial frequency and temporal changes during growth (De Gregorio et al. 2021a). These changes also involve song timing, as males show a progressively faster song tempo with age, while females become slower (De Gregorio et al. 2021a). Using this model species, we can now address the questions: Do rhythmic categories signpost an early‐emerging developmental bias? How does the development of song syntax correlate with the emergence of rhythmic categories? Do the two sexes reach adult rhythmic configuration following the same stages of development? How does the emergence of rhythmic categories correlate with their regularity around small‐integer ratios?

These developmental questions map to our 4 predictions below. The answer to each question provides insights into the biological drivers that influence the development of rhythmic production in another singing primate, and can be directly compared to developmental trends in rhythmic production in humans.

Our first prediction is that isochrony is the earliest‐emerging small‐integer ratio. Isochrony is potentially an emergent property of the nervous system, resulting from synchronizing phonation processes with neural oscillations (Kotz et al. 2018), which remain constant across developmental stages. Because of this, isochrony could represent the first basic temporal grid on which the early stages of the song are structured (Ravignani and Madison 2017).

Our second prediction is that 1:2 and 2:1 rhythmic categories appear as the song syntax develops, that is, with the refinement of song organization into phrases. Previous evidence on this species shows that the number of units emitted alone, that is, not grouped into phrases, decreases over ontogeny: in other words, the organisation of units into phrases increases gradually with age (De Gregorio et al. 2021a). The organization of units into phrases determines the alternation of shorter within‐phrase intervals and longer between‐phrases intervals (De Gregorio et al. 2021b).

Our third prediction is that the ontogenetic trajectories will be sex‐specific, with females exhibiting earlier development of the final rhythmic structure of the song. Parameters of phrase organisation and duration patterns of several features, such as individual phonation, unit durations, inter‐onset interval durations, are dimorphic in this species (De Gregorio et al. 2019b; De Gregorio et al. 2021a; Gamba et al. 2016). In addition, female indris tend to disperse before males, suggesting an earlier development of females (De Gregorio et al. 2021a), a tendency shared with other primate species (Behringer et al. 2014; Stephens and Wallen 2013).

Our fourth prediction is that adult indris will show a high degree of rhythmic regularity, that is, produced intervals will fall very close to integer ratios. In other mammal species, rhythmic regularity, quantified as the ratio between the number of rhythmic ratios falling in the vicinity versus far from isochrony, is an honest cue of the quality of the sender and is thus subject to sexual selection (Demartsev et al. 2022). Conversely, rhythmic irregularity is sometimes associated with forms of stress, reduced respiratory fitness, or genetic imperfection (Perrodin et al. 2023; Van Den Broek and Todd 2009). In indri, as in other non‐human species, singing plays a fundamental role in forming and maintaining pairs over time and is subject to sexual selection (Bonadonna et al. 2020); these selected traits should rather manifest in adulthood.

## 2 Material and Methods

### 2.1 Our Approach

We tracked the development of rhythmic categories in the songs of *Indri indri*. Indris belong to the Strepsirrhini group (our last common ancestor dates back to approximately 74 Million Years; Pozzi et al. 2014), the primate basal evolutionary branch. Our dataset provides 12 years of longitudinal song recordings from 62 individuals of both sexes of this critically endangered species endemic to the primary forests of Madagascar. First, we investigated whether the number of rhythmic categories changed across three age classes (0–2 years old, 3–4 years old and adult indris; Figure 1). Second, we tested differences in the regularity around small‐integers of rhythmic production of different age classes.

### 2.2 Animals and Recording

We monitored 12 indri groups in the Maromizaha New Protected Area (Eastern Madagascar, 18° 56′ 49′′ S, 48° 27′ 53′′ E) for 12 years, from 2010 to 2022, for a total of 63 months. We collected 839 songs from 62 identifiable individuals. All the individuals considered in this study were accustomed to the presence of researchers and did not alter their behaviour due to human presence. Twenty‐three individuals were followed through each stage of their development, from birth to dispersal or death. We recorded animals between 6:00 AM and 1:00 PM. All songs were recorded at 2–20 m distance from the animals, using various types of sound recorders (Olympus S100 and LS05, Sound Devices 702, and Tascam DR100, DR‐40, DR‐05), set at a sampling rate of 44.1 kHz, with a 16‐bit amplitude resolution (De Gregorio et al. 2021a), equipped with semi‐directional microphones (ME 67 and AKG CK 98) oriented towards the singing individual.

We divided the dataset into age classes consistent with previous literature (Rolle et al. 2021): 0–2 (up to 2.5 years old), 3–4 (from 2.6 to 4.5 years old) and adults (individuals over 4.5 years of age, including reproductive individuals). We classified individuals as juveniles up to the age of 4.5 years; this has biological and developmental significance because all females had dispersed from their natal group by the age of 4.5 years (De Gregorio et al. 2021a).

### 2.3 Acoustic Analyses

We edited and saved the songs as WAV audio files using the software Praat 6.0.56 (Boersma 2001). Based on field notes and video recordings, we identified individual contributions. We created a Praat *TextGrid* to annotate the onsets and the offsets of each note for each individual contribution to the song. We extracted all onsets of individual contribution units from multiple *TextGrids* and exported them into a *.csv* data sheet using Python (Van Rossum et al. 1995).

We calculated the inter‐onset intervals (*t_k_*, Figure 1) as the interval between a note's onset and the following note's onset. We focused just on *t_k_* ≤ 5 s because, in humans and based on available data on other primates (Honing 2018; Kuhl and Padden 1983; London 2012), it is considered the upper limit for meter perception. The rhythmic ratio (*r_k_*) was calculated by dividing each *t_k_* by its duration plus the duration of the following interval (Roeske et al. 2020): *r_k_* = *t_k_* /(*t_k_* + *t_k_*_+1_). If we imagine a sliding window proceeding along the song, the *r_k_* defines the local relationship between two adjacent *t_k_* intervals. Rhythmic ratio values provide a quantification of the overall temporal organization, allowing us to test if specific relationships (e.g., 1:1, two intervals of the same duration) are more frequent than others.

### 2.4 Statistical Analyses

#### 2.4.1 Testing Peak Significance

**FIGURE 2:**
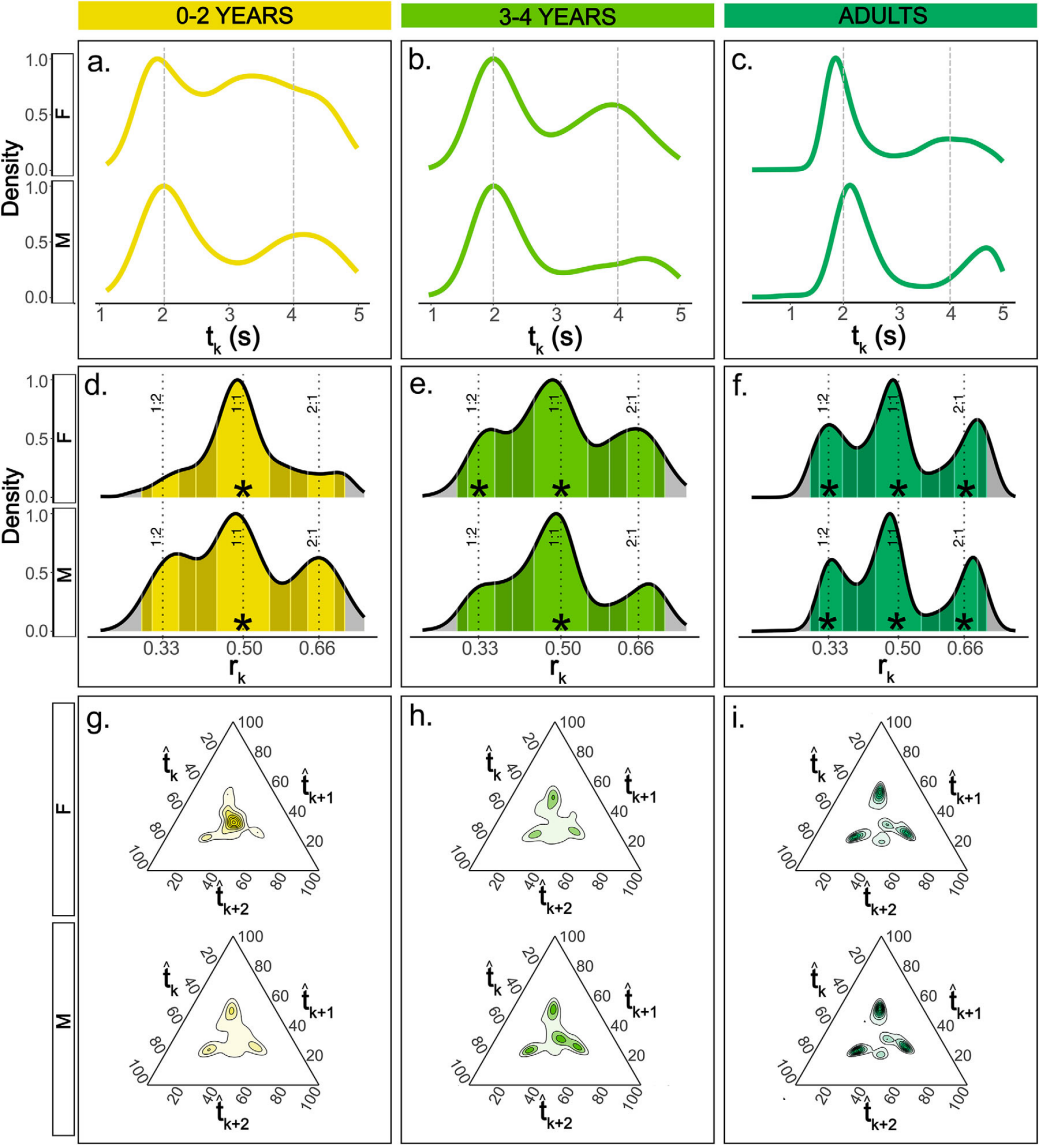
Rhythmic features of the indri's song from birth to adulthood. (a–c) Probability density function representing the distributions of *t_k_* per age and sex (the horizontal axis shows event duration in seconds). The grey dashed line indicates reference values of 2 and 4 s. (d–f) Probability density function representing the distributions of rhythm ratios (*r_k_*) per age and sex. Light‐coloured sections of the curves indicate on‐integer *r_k_* ranges, darker‐coloured sections indicate off‐integer *r_k_* ranges. Asterisks indicate significant peaks around small‐integer ratios: in the early stages of singing (0–2 years), young indris produce only isochronous patterns. The complete rhythmic configuration (1:2, 1:2, 2:1) occurs exclusively in adulthood, in both sexes. (g–i) Ternary plots for age and sex representing the duration proportions, on a scale from 0 to 100, of 3 successive *t̂_k_*. Each *t̂_k_* represents the proportion (*t̂_k_*_=_ *t_k_*/(*t_k _* + *t_k_*_+1_ + *t_k_*_+2_) of each interval composing the considered sliding window.

We tested whether peaks in the *r_k_* density distribution significantly fell around small‐integer ratios using a previously published methodology (Roeske et al. 2020). For each rhythmic category, we defined an on‐integer ratio range centred around a small‐integer ratio, and two adjacent off‐integer ratio ranges. We centred the on‐integer ratio ranges around 1:2 (or 0.333, a small integer ratio), 1:1 (or 0.500, corresponding to isochrony), and 2:1 (or 0.666, a small integer ratio). A 1:1 ratio means that two adjacent intervals have equal duration. A 1:2 ratio means that an interval has double the duration of the preceding one; conversely, a 2:1 ratio means that an interval has half the duration of the preceding one. For the 1:2 categorical rhythm, the boundaries of the on‐integer ratio were 1:3.25 (or 0.308), 1:2.75 (or 0.364), for isochrony 1:2.25 (or 0.444), 1‐1:2.25 (or 0.555), and the 2:1 is between 1‐1:2.75 (or 0.637), and 1‐1:3.25 (or 0.693). Off‐integer ratio ranges were centred around 1:3.5 (or 0.285), 1:2.5 (or 0.400), 1‐1:2.5 (or 0.600), and 1‐1:3.5 (or 0.710). For the 1:2 rhythmic category, the left‐side off‐integer ratio range was defined from 1/3.5 (or 0.287) to 1:3.25 (or 0.308), and the right‐side range from 1:2.75 (or 0.364) to 1:2.5 (or 0.400). For the 1:1 rhythmic category, the left‐side off‐integer ratio range was defined from 1/2.5 (or 0.400) to 1:2.25 (or 0.444), and the right‐side range from 1‐1:2.5 (or 0.556) to 1‐1:2.25 (or 0.600). For the 2:1 rhythmic category, the left‐side off‐integer ratio range was defined from 1‐1:2.25 (or 0.600) to 1‐1:2.75 (or 0.636), and the right‐side range from 1‐1:3.25 (or 0.692) to 1‐1/3.5 (or 0.714). All on‐ and off‐integer ratio ranges are shown in the density plots of Figure 2, with on‐integer ratio ranges indicated by lighter shaded bands and off‐integer ratio ranges by darker shaded bands. We then counted the number of *r_k_* datapoints falling in each on‐ and off‐integer ratio range per individual contribution (De Gregorio et al. 2021).

We used GLMMs to test if indris significantly produced more on‐integer than off‐integer ratios near the three peaks of the *r_k_* density distribution (Brooks et al. 2017). We ran three GLMMs, one for each age class (0–2, 3–4, adult), fitting a Poisson distribution (count variable). Our response variable was the *r_k_* count, that is, the number of rhythmic ratios falling in a specific bin. Its distribution was assessed via the package *fitdistrplus* (Delignette‐Muller et al. 2023). Our fixed factors were the particular bin in which the *r_k_* fell (on 1:2, off 1:2, on 1:1, off 1:1, on 2:1, off 2:1), the sex of the emitter, and their interaction. Since the on‐integer and off‐integer ratio ranges have uneven width, we also included an offset variable weighting the *r_k_* count based on the width of the bin as measured on the probability density curve (Laffi et al. 2025; Lameira et al. 2024). The individual identity was included as a random factor.

We checked our model to exclude zero inflation (package *performance*, function *check_zeroinflation*; Lüdecke et al. 2021). To test the significance of the *full* model, we compared it with a *null* model that included only the random factor and offset, using a likelihood ratio test (Dobson and Barnett 2018). We obtained *p*‐values for each predictor (R *summary* function) and performed Tukey *p*‐adjustments and pairwise comparisons (*emmeans* package; Lenth et al. 2022). The normality and homogeneity of residuals were verified by inspecting the *qqplot* and the residuals’ distribution (a function provided by R. Mundry).

#### 2.4.2 Testing Rhythmic Regularity Around Small‐Integers

We calculated rhythmic regularity for every peak around small‐integer ratios, a metric that quantifies the rhythmic ratios' proximity around a specific small‐integer ratio. Rhythmic regularity is computed as the ratio between the count of on‐integer observations and on‐ plus off‐integer observations of the individual contribution (Demartsev et al. 2022; Raimondi et al. 2023). The number of observed *r_k_* falling within each bin was weighted by multiplying it by the bin width, to account for the position of each bin on the probability density curve (Demartsev et al. 2022; Raimondi et al. 2023). A rhythmic regularity value of 1 means that all observations near a ratio fall in the on‐integer range, while a value of 0 means that these observations fall in the off‐integer range. Theoretically, we could expect three different trends: regularity increases with age, regularity decreases with age, or regularity does not change with age (high or low levels of regularity regardless of ontogeny). To answer that question, we investigated potential differences in *rhythmic regularity* through development; as above, we used two GLMMs, one for every small‐integer ratio (1:2, 1:1) detected as significant in at least two age classes of the two sexes. Rhythmic regularity, our response variable, followed a beta distribution for all three models (Delignette‐Muller and Dutang 2015). The sex of the emitter and age class, and their interaction, were the fixed factors, and the specific individual contribution was the random factor. For each of the three models, we compared the *full* to the *null* model using a likelihood ratio test to test the significance of the *full* model (Dobson and Barnett 2018). We obtained *p*‐values for each predictor (*summary* function), and performed *p*‐adjustment for multiple comparisons (Tukey method) and pairwise comparisons for each level of the explanatory variables (*emmeans* package; Lenth et al. 2022). The normality and homogeneity of residuals were verified by inspecting the *qqplot* and the residuals’ distribution (a function provided by R. Mundry).

#### 2.4.3 Data Visualization

To display the rhythmic structure across different age groups, we used three different representations by gradually increasing the temporal window of investigation, respectively, taking into account *one*, *two*, or *three* adjacent *t_k_* values. These three visualization methods—density plot of *t_k_*, density plot of *r_k_* and ternary plot—are complementary, showing how the rhythmic structure unfolds at different scales. The density plot of *t_k_* (Figure 2a–c) shows the overall distribution of the inter‐onset intervals (De Gregorio et al. 2021b; Roeske et al. 2020). The density plot of *r_k_* (Figure 2d–f) shows the empirical distribution of rhythmic ratios; it displays the relationships of two adjacent *t_k_* ratios clustering around specific values (i.e., the presence of categorical rhythms). If the *r_k_* distribution clusters around specific reference values (0.33 for 1:2, 0.5 for 1:1, 0.66 for 2:1, etc.), one may observe a special case of categorical rhythms, namely a small‐integer ratio rhythm. If we imagine a sliding window on a song, the ternary plots (a.k.a. simplexes; Figure 2g–i) show the rhythmic structure at a scale of three consecutive *t_k_* values (De Gregorio et al. 2024b; Jacoby and McDermott 2017). Three consecutive *t_k_* are represented by a point in a three‐dimensional space; the *x*, *y* and *z* coordinates correspond to the first, second and third intervals, respectively. The ternary plot thus represents the relative proportions, scaled to 100, of the three *t_k_* on the triangle sides or axes. This is relevant as it hints at the development of the combinatorial organisation of the songs in terms of grouping single units into phrases and alternating long (between phrases) and short (within) intervals. Table S1 and Figure S1 of the Supplementary Materials provide further details on data visualization and the interpretation of ternary plots.

## 3 Results

### 3.1 Descriptive Statistics of the *t_k_* Distribution

The distribution of the inter‐onset intervals showed two peaks at all ages for both males and females (Figure 2a–c). In females, we found *t_k_* peak values of 1.923 s (intra‐phrase) and 3.277 s (inter‐phrase) for the 0–2 years age class, values of 2.029 and 3.898 s for the 3–4 years age class, and 1.854 and 3.943 s in adults. In males, we found *t_k_* peak values of 2.003 and 3.928 s for the 0–2 age class, values of 2.046 and 4.402 s for 3–4 years of age males, and 2.156 and 4.750 s in adults.

### 3.2 Descriptive Statistics of the *r_k_* Distribution

The probability density function of *r_k_*, deriving from two adjacent *t_k_*, showed distributions that differed by sex and age group. In both sexes, as age progresses, there was an increasing density of rhythmic ratios around values corresponding to the small‐integer ratios of 1:1, 1:2 and 2:1 (Figure 2d–f). The peak around 1:1 was prominent early in development, although three peaks can be identified at all ages. In females, the peak values were 0.399 (corresponding to 1:2), 0.487 (1:1) and 0.601 (2:1) for the 0–2 age class, 0.356 (1:2), 0.483 (1:1) and 0.654 (2:1) for the 3–4 age class, and 0.331 (1:2), 0.487 (1:1) and 0.691 (2:1) for adults.

### 3.3 Significance of Peaks Around Small‐Integer Ratios

We tested if the indris significantly produced more on‐integer than off‐integer rhythmic ratios around the 1:1, 1:2 and 2:1 categories in the three age classes. Females exhibited isochrony (1:1) at all ages (Tables S2–S4), and, from age 3–4 years, the 1:2 peak became significant (Table S3). The 1:1 category was significant in males from the first developmental stage, while the complete rhythmic pattern –1:1, 1:2 and 2:1– emerged only at adult age (Tables S2–S4). All three rhythmic categories were significantly on‐integer in adult males and females (Table S4). To summarize, isochrony (1:1) was significant in both sexes from early song development. Females started to exhibit a second categorical rhythm (1:2) at 3–4 years of age. Both sexes only showed a full‐fledged rhythmic pattern –1:1, 1:2 and 2:1– in adulthood. In brief, indris’ songs showed changes in small‐integer ratios throughout ontogeny and in both sexes.

### 3.4 Distribution of Three Adjacent *t_k_*

In the ternary plots (Figure 2g–i), we displayed the relationship among three adjacent *t_k_*, each corresponding to one of the triangle's edges. Males’ rhythmic pattern seemed overall conserved during ontogeny, although the observations’ density corresponding to 2:2:1 and 1:1:1 seemed to become more defined with age. Conversely, in females, 0–2 years old showed a cluster of datapoints around isochrony (1:1:1), while 3–4 years old individuals showed a pattern similar to adults. Commonalities between sexes included a high density of points around 1:1:1, 1:1:2, 1:2:1, 2:1:1 and 2:2:1, meaning that three adjacent *t_k_* intervals typically followed one of these five patterns. Overall, these results indicate that the structure of phrases becomes increasingly more defined with age.

### 3.5 Rhythmic Regularity Around Small‐Integers

**FIGURE 3:**
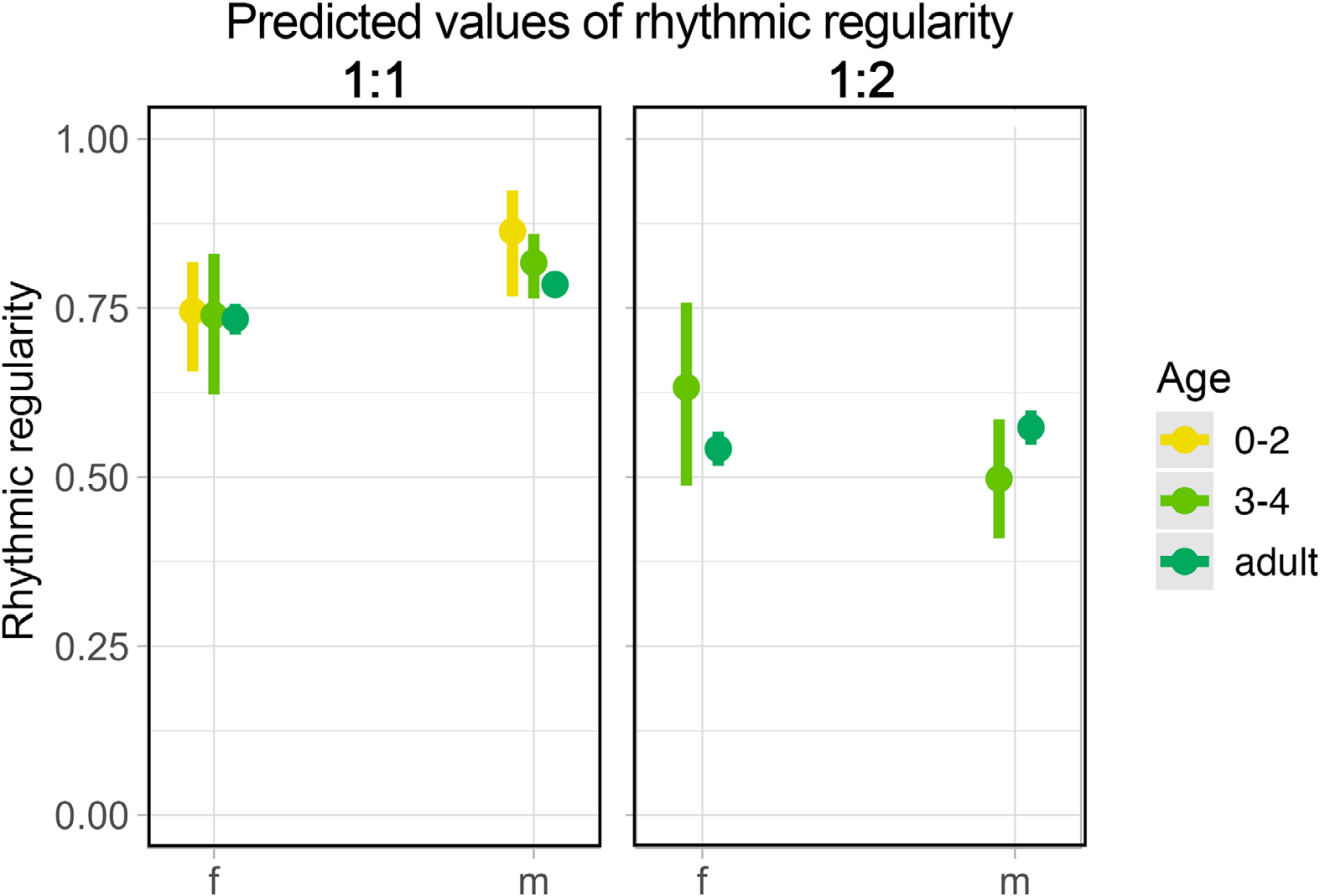
Effects plots showing the predicted values of *rhythmic regularity around small‐integers*, quantified as the proportion of on/off‐integer (1:1 and 1:2) rhythmic ratios in a single individual contribution, based on age and sex of the emitter. The models showed a significant difference in regularity between adult females and males on the 1:1 ratio (Supplementary Material S6).

For each small‐integer ratio, after testing whether the data contained a peak significantly close to it, we quantified how precise the peak was around said small‐integer ratio. *Rhythmic regularity* around isochrony did not change with development: no differences were detected among age classes. On the contrary, we found a difference between adult females and adult males’ *rhythmic regularity*, with higher regularity in males (Table S5; Figure 3). For 1:2 small‐integer ratios, the *full* models were not significantly different from the *null* models, suggesting that the rhythmic regularity of 1:2 small‐integer production does not change during ontogeny. To recapitulate, although the occurrence of rhythmic categories corresponding to different small‐integer ratios depends on both sex and age, the regularity around these categories is the same across age groups.

## 4 Discussion

### 4.1 Overview

We mapped how the distribution of rhythmic categories changes during ontogeny in a non‐human primate song. The isochronous rhythmic pattern (1:1) is present from the earliest stages of song development in both sexes. In contrast, two other small‐integer ratios (1:2 and 2:1) follow sex‐specific developmental trajectories. Only in adulthood indris show all three small‐integer ratios, 1:2, 1:1 and 2:1.

### 4.2 Isochrony Is Characteristic of Indris’ Song

We found that isochrony is present in songs of both sexes of all ages, in agreement with our first prediction. Isochronous patterns are plausibly linked to neural and physiological mechanisms shared among a broad range of animal species (Wilson and Cook 2016). Since periodic neural oscillations can be observed in many species and at different ages, it does not seem surprising that indris, like Cape fur seal pups (Osiecka et al. 2024) and children (Nave et al. 2024), produce isochronous sequences from the early stages of development. This early‐emerging temporal regularity may reflect conserved biological constraints shared at least across mammals, including humans. Why then do many species not produce any rhythmic categories or isochrony at all? Recent comparative efforts have shown that isochrony in spontaneous vocal production is anecdotally found in several mammalian vocalisations but is far from universal. For instance, harbour seal pup calls (Anichini et al. 2023) do not contain any rhythmic categories. Even other vocal types in indris, such as *roars* emitted before songs or as an alarm for aerial predators (De Gregorio et al. 2024), lack basic isochrony. In other words, not all vocal tokens are produced on an isochronous grid, highlighting how periodicity can be adaptively deployed depending on the function of a signal.

If some of the selective pressures acting on the early vocal output of the human evolutionary branch and on the non‐human primate song are similar, comparing rhythmic development in humans and indris may help identify shared evolutionary roots. In humans as in indris, isochrony is a dominant pattern from the early stages of development (Bobin‐Bègue 2019). Infants spontaneously produce isochronous patterns in their motor (e.g., sucking, limb movements) and vocal (e.g., crying, babbling) behaviours (Bobin‐Bègue 2019; Provasi et al. 2014; Tafuri and Villa 2002), and exhibit sensitivity to isochronous patterns very early in development (before one year of age; Hannon et al. 2018; Zentner and Eerola 2010). Also, children synchronise better with an isochronous sequence than with a more complex rhythm pattern (Hannon et al. 2018; Reifinger 2006), and show early affinity for regular beats, one of the few early‐developing universal constraints for music acquisition (Hannon and Trainor 2007). Even though culture‐specific experience seems to play a prominent role in shaping the development of rhythm processing and production in humans, there is some evidence that ratio complexity per se constrains rhythm perception even before the acquisition of culture‐specific biases (Hannon et al. 2011). Unfortunately, we have no comparable data on rhythm perception in young indris, and we can just infer about the rhythm production. Isochrony may represent a step in developing rhythmic capacities, serving as a building block for more complex rhythmic abilities. Proto‐musical behaviours in the hominoid evolutionary branch (Merker et al. 2009) share a set of key behavioural and functional features with the nonhuman‐primate song, as a choral context of emission and an advertisement purpose (De Gregorio et al. 2022; Geissmann 1999). In other words, our hominoid ancestors and other primate species could have experienced similar selective pressures during the evolution of their collective vocalizations (Geissmann 1999; Merker et al. 2009).

### 4.3 Indris Produce Increasingly More Rhythmic Categories Over the Lifespan

Our second prediction was that the additional small‐integer ratios (1:2, 2:1) observed in adults would not appear immediately, but in later stages of an individual's development. Our data confirmed this, showing the emergence of 1:2 and 2:1 rhythmic categories at different stages of song development. In particular, young males only exhibited isochrony at 1–2 and 3–4 years of age, while young females consistently exhibited isochrony and additionally displayed the 1:2 category starting from 3–4 years of age. It is unclear whether these developmental changes occur gradually or with sudden changes, either during the 3–4 year age period in females or at the onset of adulthood in males.

Recent work on rhythm categories in children has spurred comparative researchers to study vocal rhythm production in other animal species. Human musical systems are based on interval discretisation, meaning that the emitted intervals are not randomly distributed but clustered around specific values (Brown and Jordania 2013; Savage et al. 2015). Also, their relationships converge around small‐integer ratios (Roeske et al. 2020; Savage et al. 2015), a trait that is widely shared across all cultures (Jacoby et al. 2024).

In children's iterative rhythm production tasks, consisting of tapping to synchronise to repeated intervals chosen randomly, the incidence of the 2:2:3 and 2:3:3 three‐intervals relationships increases significantly with age (Nave et al. 2024). The ability to reproduce and synchronise with rhythms more complex than isochrony seems to be gradually acquired and refined during development as well as influenced by culture‐specific listening experiences (Hannon et al. 2012; Hannon and Trainor 2007; Jacoby and McDermott 2017; Ullal‐Gupta et al. 2014). Importantly, ratio complexity constrains rhythm perception *before* the integration of culture‐specific biases (Hannon et al. 2011). These tasks require the integration of typically human perception and production modules: for instance, the organisation into simple ratios is interlinked with categorical perception, in which the intervals between signals are interpreted as discrete elements (Desain and Honing 2003; Sadakata et al. 2006). On the other hand, the development of spontaneous rhythm production in infants’ repertoires may offer a glimpse into fundamental mechanisms that allow the mere production of increasingly complex rhythms with development across species. Overall, evidence shows that children display spontaneous rhythmic features that complexify throughout development (Bobin‐Bègue 2019). Crying and babbling, the ontogenetic precursors to rhythmic skills that precede musicality and speech (Oller 2000; Stadler Elmer 2020), become more complex with age (Blake and Boysson‐Bardies 1992; Morgan and Wren 2018; Oller 2000; Provasi et al. 2014).

The ability to gradually structure communicative signals on more complex rhythms has a crucial adaptive value for many species' social dynamics (Lipkind et al. 2013). Flexible synchrony and turn‐taking in social interactions are essential for communication, increasing or avoiding overlap, expressing emotion, and improving social bonds, in humans (Bowling et al. 2013; Pouw and Holler 2022) as well as in other animal species (e.g., de Reus et al. 2020; Geissmann and Orgeldinger 2000; Ravignani 2019). Perhaps because of this adaptive advantage, in most mammals, signal structure complexifies with development. Structural rhythmic changes during ontogeny occur in many species and are closely intertwined with the signal's combinatoriality (Lipkind et al. 2013). The combinatorial characteristics of the signal go hand in hand with the degree of its structural and semantic complexity. Most species receive an adaptive advantage in developing an adult signal structure that allows encoding more information by concatenating elements in a series (Engesser and Townsend 2019). In the development of children's and other animal babbling, for example, in passerine song, early vocal production gradually integrates new combinatorial elements and the transitions between them (Fernandez et al. 2021; Langehennig‐Peristenidou et al. 2023; Lipkind et al. 2013). In short, introducing new element types of different durations and transitions between elements determines the emergence of new rhythmic patterns in development (Lipkind et al. 2013; Nave et al. 2024).

Rhythmic combinatoriality is a fundamental aspect of indris’ rhythmic patterns: indris' singing undergoes radical changes based on age, sex, and status in how the rhythms are structured (Cristiano et al. 2023; De Gregorio et al. 2021a; De Gregorio et al. 2025c; Zanoli et al. 2020, 2023). Previous evidence suggested that small‐integer ratios in indris’ singing are linked to the song's structure, which is organised into phrase units (De Gregorio et al. 2021b). Local isochrony (1:1) emerges from consecutive intervals within a phrase. At the same time, the binary (2:1 and 1:2) categories (De Gregorio et al. 2024) derive from the alternation of long (between phrases) and short (within a phrase) intervals (De Gregorio et al. 2021a). Indris of the same sex (mother and daughter or father and son) show scarce overlapping in a chorus (Gamba et al. 2016): compared to the adult's contribution, a juvenile's song will show longer silences (even on the scale of several minutes) between groups of phrases. This is not the case for adults, where 1:2 and 2:1 small‐integers derive from the rapid alternation of short (within‐phrase) and long (between‐phrases) intervals in adults (De Gregorio et al. 2021b). The different song architecture of adults and juveniles, and thus the smaller number of transitions between phrases in the latter, can explain the absence of the 1:2 and 2:1 categories at birth. Finally, adult indris increase the duration of units as the phrase progresses (Valente et al. 2021). In other words, we can expect the *t_k_* of the last element of each phrase to be longer, determining the slight right shift around the 2:1 small‐integer observed in both sexes. This shift may also explain why females of 3–4 years of age produce 1:2 rhythmic categories, but not 2:1. In several animal species, the final elements of an utterance are, indeed, longer (Huang et al. 2020).

The mechanisms responsible for increasing the number of observed rhythmic categories during development are potentially numerous. Here, combinatoriality seems to play a key role. Breathing and physiology could also indirectly spur new rhythmic categories in indri. In marmosets, maturational changes during growth affect vocal development and signal structure (Pistorio et al. 2006; Zhang and Ghazanfar 2018): control of respiration coupled with development of the vocal apparatus may affect the rhythmic patterns produced at different stages of development (Leonetti et al. 2024). We confirmed that the rhythmic structure of the indris’ song changes with development, in line with previous evidence on the vocal ontogeny of Strepsirrhine infant vocal streams (Langehennig‐Peristenidou et al. 2023) and indris’ song (De Gregorio et al. 2021a). We observed an increase in rhythmic complexity with age, though the specific, potentially synergistic, underlying mechanisms remain unclear.

### 4.4 Rhythmic Features Follow Sex‐Specific Developmental Trajectories

We found distinct rhythmic structures between males and females (Figure 2). In both sexes, a complete rhythmic structure was present only in adulthood. However, in the emergence of rhythmic categories, females already showed the 1:2 category at 3–4 years, while males only showed this small‐integer ratio as adults. Our results confirmed our third prediction, that the developmental trajectories would follow sex‐specific trends. These findings align with previous results showing that durational parameters (cumulative phonation, contribution, inter‐phrase inter‐onset intervals and unit duration) follow opposite dimorphic trajectories throughout development, and that indris’ song rhythmicity is influenced by sex‐specific factors (De Gregorio et al. 2019a, De Gregorio et al. 2019b). Previous findings on this species (De Gregorio et al. 2021a) demonstrated that females around 1–2 years old frequently use single notes, that is, units emitted alone, not embedded into more extensive phrases. The frequent use of consecutive single notes explains why the 1:1:1 pattern of three consecutive *t_k_* was so pronounced in females of 0–2 years of age, while it was less clear in the sporadic use of single notes in males.

Notably, such dimorphism in the development of song rhythmicity cannot result from morphological sexual dimorphism: females and males show practically no difference in the mass and size of their vocal sac (Dixson 1998; Pollock 1986). Indeed, in lemurids a shortened growth period has been proposed to represent the limit to the emergence of adult morphological sexual dimorphism (Leigh and Terranova 1998). Consistent with this pattern, indris do not exhibit marked sexual dimorphism in morphology, yet they show pronounced dimorphism in vocal behaviour (e.g., Giacoma et al. 2010). Given that sexually dimorphic traits are typically linked to divergent developmental trajectories between males and females (Setchell and Lee 2004), the presence of sex‐specific developmental patterns in indri vocal behaviour is therefore consistent with general patterns observed in primates. Moreover, females appear to reach maturity earlier than males, which could explain the emergence of additional rhythmic categories beyond isochrony in females before they are observed in males (De Gregorio et al. 2021a). We can speculate that the observed sex differences in the song features are linked to sexual dimorphism in the neurobiological mechanisms responsible for song processing and production. More rhythmic categories in younger females echo higher flexibility in adjusting the song's rhythmic structure to overlap with chorusing males (Giacoma et al. 2010; Zanoli et al. 2020, 2023). Our findings thus support the presence of a developmental sexual dimorphism, as males and females showed distinct developmental trajectories, with both sexes producing complete rhythmic patterns after reaching sexual maturity.

### 4.5 Indris Do Not Refine Rhythmic Regularity With Age

Finally, we predicted a positive correlation between age and *rhythmic regularity* around small‐integer ratios. Rhythmic regularity was not different across age classes. This is in contrast with our last prediction and partially unexpected because young indris show higher variability of between‐phrases intervals than adults (De Gregorio et al. 2021a).

In the indris, the regularity in the produced isochrony (1:1) did not change with age in either sex: once that ratio appeared in development, it was as rhythmically stable as in adulthood. This is in contrast with previous evidence on another vocally active mammal producing isochronous patterns, Cape fur seals, which display higher isochrony in adults than in pups (Osiecka et al. 2024). Our results suggest a potentially innate, precise temporal tuning of isochrony in the indri song (Geissmann 1984, 1999).

When the 1:2 small‐integer ratio appears in females of 3–4 years, it already shows a similar rhythmic regularity to that observed in adults, with no evidence of a gradual convergence towards typical adult intervals. Instead, there is a reorganisation of the song structure: early in life, units are unorganised and not clustered into phrases, becoming more distinctly clustered in phrases with growth. Moreover, as previously noted, the number of single notes produced by females decreases significantly in the second stage of development (3–4 years), further indicating a fundamental shift in how indris structure their songs. The abrupt appearance of combinatoriality in the target song has been observed in other singing species, such as zebra finches (Lipkind et al. 2013).

Our results suggest a lack of increase in rhythmic regularity, in contrast with some evidence in humans. Children as young as six years old can produce small integer‐ratio rhythmic categories in tapping tasks: with age, tapping precision and stability increase (McAuley et al. 2006; Nave et al. 2024; Rocha et al. 2021a). How much the stability of rhythm priors, alongside the development of motor and memory capacities, contributes to these observations remains to be fully understood (Hannon et al. 2018; Monier and Droit‐Volet 2019; Nave et al. 2024).

From an evolutionary perspective, what kind of valuable information could the production of a highly regular signal mediate? In‐silico models suggest that rhythmic signals enable the greatest discrimination between high‐ and low‐quality partners, with low quality characterized by decreased rhythmic regularity in artificial signals (Van Den Broek and Todd 2003). This is mirrored in empirical evidence showing that signal rhythmic regularity is indeed subject to sexual selection and is an honest signal of the transmitter's quality (De Gregorio et al. 2025a; Van Den Broek and Todd 2009). Whether rhythmic stability predicts reproductive success in indris warrants further exploration.

### 4.6 Final Considerations

We quantified the development of vocal rhythms in a non‐human primate belonging to the early‐diverging branch of primate evolution, the indri. Our data highlight how a key feature of human music, rhythmic categories around small‐integer ratios, is present in all life phases of this species’ song. While we find some similarities between indris’ development and humans, the differences stand out. We show how rhythmic categories corresponding to small‐integer ratios follow lifespan trajectories with sex‐specific trends. In humans and other mammals, the production of complex rhythms develops with age. Similarly, in indris, rhythmic patterns are transformed and become more complex with age, resulting from song organisation that undergoes clustering during development. The comparative study of the ontogeny of rhythm is in its infancy. However, it represents a fundamental step in understanding the biological roots of musicality.

## Author Contributions

**Lia Laffi**: conceptualization, data curation, investigation, validation, writing – original draft, writing – review and editing, formal analysis, visualization, methodology. **Teresa Raimondi**: conceptualization, data curation, investigation, validation, writing – original draft, writing – review and editing, formal analysis, visualization, methodology. **Chiara De Gregorio**: conceptualization, data curation, writing – original draft, writing – review and editing. **Daria Valente, Walter Cristiano, Filippo Carugati, Valeria Ferrario, Valeria Torti**: data curation, writing – review and editing. **Jonah Ratsimbatsafy, Cristina Giacoma**: writing – review and editing. **Andrea Ravignani**: writing – original draft, writing – review and editing, supervision. **Marco Gamba**: conceptualization, visualization, supervision, writing – review and editing.

## Funding

This research was supported by the University of Torino and the Parco Natura Viva—Centro Tutela Specie Minacciate, with the financial assistance of the European Union, through the Project BIRD (ACP SandT Program, Contract FED/2009/217077). The Comparative Bioacoustics Group is supported by Max Planck Independent Research Group Leader funding to A.R. Center for Music in the Brain is funded by the Danish National Research Foundation (DNRF117), the Lundbeck Foundation (R469‐2024‐1573) and Købmand Herman Sallings Fond. L.L. is partially funded by Fondazione ZOOM. L.L., T.R. and A.R. are funded by the European Union (ERC, TOHR, 101041885). Views and opinions expressed are however those of the authors only and do not necessarily reflect those of the European Union or the European Research Council Executive Agency. Neither the European Union nor the granting authority can be held responsible for them.

## Ethics Statement

We complied with all the good practice protocols for field primatology of the International Primatological Society (2014) to work with wild species of primates. ‘Direction des Eaux et Forêts’ and ‘Madagascar National Parks’ provided us with research permits for collecting non‐invasive acoustic recordings of wild indris. We never used playback stimuli to avoid any modification of indris’ behaviour. All procedures follow the guidelines of the Association for the Study of Animal Behaviour for the care and use of animals for research activities (2024).

## Conflicts of Interest

The authors declare no conflicts of interest.

## Supporting information




**Supplementary Information**: desc70189‐sup‐0001‐SuppMat.docx

## Data Availability

Data and R codes used for this study are available from a GitHub digital repository: https://github.com/teresaraimondi/The‐ontogeny‐of‐vocal‐rhythms‐in‐a‐non‐human‐primate. Additional tables and plots are provided in Electronic Supplementary Information.
