# Supplementary material for: The Ontogeny of Vocal Rhythms in a Non‐Human Primate
Source: Dev Sci. 2026 Apr 23;29:e70189. doi: 10.1111/desc.70189 (PMC13106920; doi:10.1111/desc.70189)
Supplement: Supplementary file 1 — Supplementary Information: desc70189‐sup‐0001‐SuppMat.docx [file DESC-29-e70189-s001.docx]

**The ontogeny of vocal rhythms in a non-human primate**

Supporting Information

**Table S1 – Details on the data visualization.**

| To display the rhythmic structure across different age groups, we used three different representations by gradually increasing the temporal window of investigation, respectively, taking into account *one*, *two*, or *three* successive t_k_ values. These three visualization methods are complementary, showing how the rhythmic structure unfolds at different scales. The density plot of t_k_ (**Fig 2a-2c**) shows the overall distribution of the inter-onset intervals [24,25]. The density plot of r_k_ (**Fig 2d-2f**) shows the empirical distribution of rhythmic ratios [24,25]; it displays the relationships of two adjacent t_k_ ratios clustering around specific values (*i.e.* the presence of categorical rhythms). If the r_k_ distribution clusters around the reference values (0.33 for 1:2, 0.5 for 1:1, 0.66 for 2:1), we observe the special case of a categorical rhythm called a small-integer ratio. The ternary plots (a.k.a. simplexes; **Fig 2g-2i)** show the rhythmic structure at a scale of three consecutive t_k_ values [26]. Three consecutive t_k_ are represented by a point in a three-dimensional space; the x, y, and z coordinates correspond to the first, second, and third intervals, respectively. The ternary plot thus represents the relative proportions, scaled to 100, of the three t_k_ on the triangle sides or axes. This is relevant as it hints at the development of the combinatorial organisation of the songs in terms of grouping single units into phrases and alternating long (between phrases) and short (within) intervals. **Fig. S1** of the **Supplementary Materials** provides further details on ternary plots interpretation. |
| --- |

**Figure S1 – Ternary plot interpretation.** Ternary plots display the relationship among three successive intervals, each corresponding to one of the triangle’s edges (t_k_, t_k+1_, t_k+2_). Here we evidence seven points corresponding to 1:1:1, 1:2:1, 1:1:2, 2:1:1, 1:2:2, 2:1:2, 2:2:1 relationship.


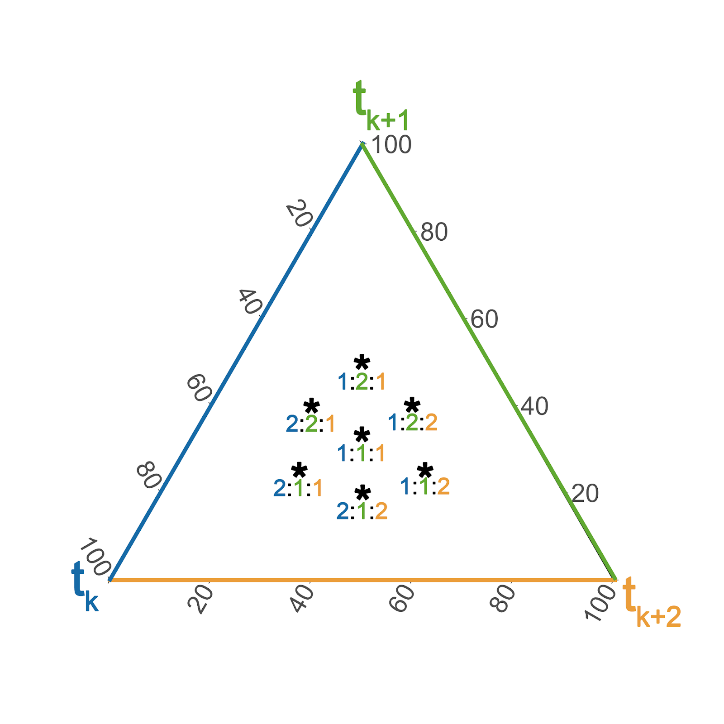


**Table S2 -** Summary and details of the GLMM and pairwise post-hoc test searching for the effect of r_k_ interval type (on-integer and off-integer for each ratio for 1:1, 1:2 and 2:1 category) and sex on the observation count (*i.e.* number of observed r_k_) for individuals aged between 0 and 2.5 years (**AGE CLASS: 0-2**).

**a -** Summary of the full model - Influence of the fixed factors (r_k_ interval: 11 on - 11off - 12on - 12off - 21on - 21off; Sex: Female - Male) on the count of the observations. The identification code of the contribution is the random factor (singer ID)

**b -** Results for the pairwise post-hoc test on the interaction r_k_ interval * sex

| **a**  **Generalized Linear Model**  Family: beta  Count (r_k_ obs) ~ r_k_ interval * sex + offset + (singer ID)  Full vs Null (Chisq= 224.527; df=11; p<.0001) | | | | | | | | | | | | | |
| --- | --- | --- | --- | --- | --- | --- | --- | --- | --- | --- | --- | --- | --- |
| **Variable** | | | | **Estimate** | | | **SE** | | | **z value** | | **p-value (z)** | |
| (Intercept) ^b,c^ | | | | 0.343 | | | 0.200 | | | ^a^ | | ^a^ | |
| r_k_ interval (11on) ^b,c^ | | | | 1.003 | | | 0.131 | | | 7.657 | | <.0001 | |
| r_k_ interval (12off) ^b,c^ | | | | -0.966 | | | 0.244 | | | -3.965 | | <.0001 | |
| r_k_ interval (12on) ^b,c^ | | | | -0.853 | | | 0.246 | | | -3.470 | | 0.001 | |
| r_k_ interval (21off) ^b,c^ | | | | -0.637 | | | 0.217 | | | -2.940 | | 0.003 | |
| r_k_ interval (21on) ^b,c^ | | | | -0.836 | | | 0.232 | | | -3.606 | | <.001 | |
| sex (m) ^b,c^ | | | | -0.582 | | | 0.382 | | | -1.522 | | 0.128 | |
| r_k_ interval (11on): sex (m) ^b,c^ | | | | 0.803 | | | 0.321 | | | 2.505 | | 0.012 | |
| r_k_ interval (12off): sex (m) ^b,c^ | | | | 1.728 | | | 0.422 | | | 4.094 | | <.0001 | |
| r_k_ interval (12on): sex (m) ^b,c^ | | | | 1.850 | | | 0.414 | | | 4.473 | | <.0001 | |
| r_k_ interval (21off): sex (m) ^b,c^ | | | | 1.142 | | | 0.421 | | | 2.713 | | 0.007 | |
| r_k_ interval (21on): sex (m) ^b,c^ | | | | 1.945 | | | 0.403 | | | 4.820 | | <.0001 | |
| ^a^ Not shown as not having a meaningful interpretation  ^b^ Estimate ± SE refer to the difference of the response between the reported level of this categorical predictor and the reference category of the same predictor  ^c^ Reference categories: “r_k_ interval (11off)”, “sex (f)” | | | | | | | | | | | | | |
| **b**  ***Post-hoc* comparisons** | | | | | | | | | | | | | |
| **Emmeans** | | | | | | | | | | | | | |
| **r_k_ interval** | **sex** | | **emmean** | | | **SE** | | | **lower.CL** | | | | **upper.CL** |
| 11off | f | | 0.414 | | | 0.200 | | | 0.022 | | | | 0.807 |
| 11on | f | | 1.418 | | | 0.175 | | | 1.075 | | | | 1.761 |
| 12off | f | | -0.552 | | | 0.272 | | | -1.085 | | | | -0.019 |
| 12on | f | | -0.439 | | | 0.274 | | | -0.976 | | | | 0.098 |
| 21off | f | | -0.222 | | | 0.248 | | | -0.708 | | | | 0.263 |
| 21on | f | | -0.421 | | | 0.261 | | | -0.932 | | | | 0.089 |
| 11off | m | | -0.167 | | | 0.329 | | | -0.813 | | | | 0.478 |
| 11on | m | | 1.639 | | | 0.210 | | | 1.228 | | | | 2.051 |
| 12off | m | | 0.594 | | | 0.278 | | | 0.049 | | | | 1.139 |
| 12on | m | | 0.829 | | | 0.264 | | | 0.313 | | | | 1.346 |
| 21off | m | | 0.338 | | | 0.301 | | | -0.252 | | | | 0.928 |
| 21on | m | | 0.942 | | | 0.263 | | | 0.426 | | | | 1.457 |
| **Contrasts** | | | | | | | | | | | | | |
| **Contrast** | | **Estimate** | | | **SE** | | | **t ratio** | | | **p-value** | | |
| **11off f - 11on f** | | **-1.003** | | | **0.131** | | | **-7.657** | | | **<.0001** | | |
| 11off f - 12off f | | 0.966 | | | 0.244 | | | 3.965 | | | 0.004 | | |
| 11off f - 12on f | | 0.853 | | | 0.246 | | | 3.470 | | | 0.026 | | |
| 11off f - 21off f | | 0.637 | | | 0.217 | | | 2.940 | | | 0.127 | | |
| 11off f - 21on f | | 0.836 | | | 0.232 | | | 3.606 | | | 0.016 | | |
| 11off f - 11off m | | 0.582 | | | 0.382 | | | 1.522 | | | 0.935 | | |
| 11off f - 11on m | | -1.225 | | | 0.289 | | | -4.240 | | | 0.001 | | |
| 11off f - 12off m | | -0.180 | | | 0.340 | | | -0.528 | | | 1.000 | | |
| 11off f - 12on m | | -0.415 | | | 0.329 | | | -1.263 | | | 0.984 | | |
| 11off f - 21off m | | 0.077 | | | 0.359 | | | 0.213 | | | 1.000 | | |
| 11off f - 21on m | | -0.527 | | | 0.328 | | | -1.608 | | | 0.907 | | |
| 11on f - 12off f | | 1.970 | | | 0.228 | | | 8.644 | | | <.0001 | | |
| 11on f - 12on f | | 1.857 | | | 0.231 | | | 8.035 | | | <.0001 | | |
| 11on f - 21off f | | 1.640 | | | 0.198 | | | 8.287 | | | <.0001 | | |
| 11on f - 21on f | | 1.839 | | | 0.215 | | | 8.564 | | | <.0001 | | |
| 11on f - 11off m | | 1.585 | | | 0.371 | | | 4.274 | | | 0.001 | | |
| 11on f - 11on m | | -0.222 | | | 0.272 | | | -0.816 | | | 1.000 | | |
| 11on f - 12off m | | 0.824 | | | 0.328 | | | 2.515 | | | 0.331 | | |
| 11on f - 12on m | | 0.588 | | | 0.315 | | | 1.868 | | | 0.779 | | |
| 11on f - 21off m | | 1.080 | | | 0.347 | | | 3.110 | | | 0.080 | | |
| 11on f - 21on m | | 0.476 | | | 0.314 | | | 1.514 | | | 0.937 | | |
| **12off f - 12on f** | | **-0.113** | | | **0.294** | | | **-0.384** | | | **1.000** | | |
| 12off f - 21off f | | -0.330 | | | 0.275 | | | -1.200 | | | 0.989 | | |
| 12off f - 21on f | | -0.131 | | | 0.286 | | | -0.456 | | | 1.000 | | |
| 12off f - 11off m | | -0.385 | | | 0.419 | | | -0.917 | | | 0.999 | | |
| 12off f - 11on m | | -2.191 | | | 0.343 | | | -6.396 | | | <.0001 | | |
| 12off f - 12off m | | -1.146 | | | 0.382 | | | -2.999 | | | 0.109 | | |
| 12off f - 12on m | | -1.381 | | | 0.373 | | | -3.704 | | | 0.012 | | |
| 12off f - 21off m | | -0.890 | | | 0.398 | | | -2.237 | | | 0.523 | | |
| 12off f - 21on m | | -1.494 | | | 0.371 | | | -4.021 | | | 0.003 | | |
| 12on f - 21off f | | -0.217 | | | 0.274 | | | -0.790 | | | 1.000 | | |
| 12on f - 21on f | | -0.018 | | | 0.285 | | | -0.062 | | | 1.000 | | |
| 12on f - 11off m | | -0.272 | | | 0.420 | | | -0.648 | | | 1.000 | | |
| 12on f - 11on m | | -2.078 | | | 0.344 | | | -6.033 | | | <.0001 | | |
| 12on f - 12off m | | -1.033 | | | 0.382 | | | -2.702 | | | 0.225 | | |
| 12on f - 12on m | | -1.269 | | | 0.374 | | | -3.395 | | | 0.033 | | |
| 12on f - 21off m | | -0.777 | | | 0.398 | | | -1.953 | | | 0.725 | | |
| 12on f - 21on m | | -1.381 | | | 0.372 | | | -3.712 | | | 0.011 | | |
| **21off f - 21on f** | | **0.199** | | | **0.265** | | | **0.751** | | | **1.000** | | |
| 21off f - 11off m | | -0.055 | | | 0.405 | | | -0.136 | | | 1.000 | | |
| 21off f - 11on m | | -1.862 | | | 0.324 | | | -5.752 | | | <.0001 | | |
| 21off f - 12off m | | -0.816 | | | 0.366 | | | -2.230 | | | 0.527 | | |
| 21off f - 12on m | | -1.052 | | | 0.356 | | | -2.952 | | | 0.123 | | |
| 21off f - 21off m | | -0.560 | | | 0.383 | | | -1.464 | | | 0.950 | | |
| 21off f - 21on m | | -1.164 | | | 0.355 | | | -3.280 | | | 0.048 | | |
| 21on f - 11off m | | -0.254 | | | 0.413 | | | -0.616 | | | 1.000 | | |
| 21on f - 11on m | | -2.061 | | | 0.334 | | | -6.173 | | | <.0001 | | |
| 21on f - 12off m | | -1.015 | | | 0.375 | | | -2.710 | | | 0.221 | | |
| 21on f - 12on m | | -1.251 | | | 0.365 | | | -3.425 | | | 0.030 | | |
| 21on f - 21off m | | -0.759 | | | 0.391 | | | -1.943 | | | 0.732 | | |
| 21on f - 21on m | | -1.363 | | | 0.364 | | | -3.746 | | | 0.010 | | |
| **11off m - 11on m** | | **-1.807** | | | **0.295** | | | **-6.130** | | | **<.0001** | | |
| 11off m - 12off m | | -0.761 | | | 0.344 | | | -2.212 | | | 0.541 | | |
| 11off m - 12on m | | -0.997 | | | 0.330 | | | -3.018 | | | 0.103 | | |
| 11off m - 21off m | | -0.505 | | | 0.362 | | | -1.397 | | | 0.965 | | |
| 11off m - 21on m | | -1.109 | | | 0.329 | | | -3.366 | | | 0.037 | | |
| 11on m - 12off m | | 1.045 | | | 0.237 | | | 4.412 | | | 0.001 | | |
| 11on m - 12on m | | 0.810 | | | 0.215 | | | 3.771 | | | 0.009 | | |
| 11on m - 21off m | | 1.302 | | | 0.263 | | | 4.950 | | | <.0001 | | |
| 11on m - 21on m | | 0.698 | | | 0.215 | | | 3.246 | | | 0.054 | | |
| **12off m - 12on m** | | **-0.236** | | | **0.281** | | | **-0.839** | | | **1.000** | | |
| 12off m - 21off m | | 0.256 | | | 0.317 | | | 0.809 | | | 1.000 | | |
| 12off m - 21on m | | -0.348 | | | 0.280 | | | -1.242 | | | 0.986 | | |
| 12on m - 21off m | | 0.492 | | | 0.302 | | | 1.627 | | | 0.899 | | |
| 12on m - 21on m | | -0.112 | | | 0.261 | | | -0.429 | | | 1.000 | | |
| **21off m - 21on m** | | **-0.604** | | | **0.301** | | | **-2.003** | | | **0.692** | | |

**Table S3 -** Summary and details of the GLMM and pairwise post-hoc test searching for the effect of r_k_ interval type (on-integer and off-integer for each ratio for 1:1, 1:2 and 2:1 category) and sex on the observation count (*i.e.* number of observed r_k_) for individuals aged between 2.6 and 4.5 years (**AGE CLASS: 3-4**).

**a -** Summary of the full model - Influence of the fixed factors (r_k_ interval: 11 on - 11off - 12on - 12off - 21on - 21off; Sex: Female - Male) on the count of the observations. The identification code of the contribution is the random factor (singer ID)

**b -** Results for the pairwise post-hoc test on the interaction r_k_ interval * sex

| **a**  **Generalized Linear Model**  Family: beta  Count (r_k_ obs) ~ r_k_ interval * sex + offset + (singer ID)  Full vs Null (Chisq=289.569; df=11; p<.0001) | | | | | | | | | | | | | |
| --- | --- | --- | --- | --- | --- | --- | --- | --- | --- | --- | --- | --- | --- |
| **Variable** | | | | **Estimate** | | | **SE** | | | **z value** | | **p-value (z)** | |
| (Intercept) ^b,c^ | | | | 0.474 | | | 0.221 | | | ^a^ | | ^a^ | |
| r_k_ interval (11on) ^b,c^ | | | | 0.714 | | | 0.168 | | | 4.241 | | <.001 | |
| r_k_ interval (12off) ^b,c^ | | | | -1.004 | | | 0.271 | | | -3.701 | | <.001 | |
| r_k_ interval (12on) ^b,c^ | | | | -0.048 | | | 0.209 | | | -0.228 | | 0.819 | |
| r_k_ interval (21off) ^b,c^ | | | | -0.435 | | | 0.222 | | | -1.955 | | 0.051 | |
| r_k_ interval (21on) ^b,c^ | | | | -0.134 | | | 0.208 | | | -0.644 | | 0.519 | |
| sex (m) ^b,c^ | | | | -0.290 | | | 0.287 | | | -1.009 | | 0.313 | |
| r_k_ interval (11on): sex (m) ^b,c^ | | | | 0.360 | | | 0.205 | | | 1.757 | | 0.079 | |
| r_k_ interval (12off): sex (m) ^b,c^ | | | | 0.658 | | | 0.314 | | | 2.098 | | 0.036 | |
| r_k_ interval (12on): sex (m) ^b,c^ | | | | -0.128 | | | 0.258 | | | -0.497 | | 0.619 | |
| r_k_ interval (21off): sex (m) ^b,c^ | | | | -0.099 | | | 0.277 | | | -0.359 | | 0.720 | |
| r_k_ interval (21on): sex (m) ^b,c^ | | | | -0.087 | | | 0.258 | | | -0.338 | | 0.735 | |
| ^a^ Not shown as not having a meaningful interpretation  ^b^ Estimate ± SE refer to the difference of the response between the reported level of this categorical predictor and the reference category of the same predictor  ^c^ Reference categories: “r_k_ interval (11off)”, “sex (f)” | | | | | | | | | | | | | |
| **b**  ***Post-hoc* comparisons** | | | | | | | | | | | | | |
| **Emmeans** | | | | | | | | | | | | | |
| **r_k_ interval** | **sex** | | **emmean** | | | **SE** | | | **lower.CL** | | | | **upper.CL** |
| 11off | f | | 0.545 | | | 0.221 | | | 0.111 | | | | 0.979 |
| 11on | f | | 1.259 | | | 0.194 | | | 0.878 | | | | 1.640 |
| 12off | f | | -0.459 | | | 0.291 | | | -1.029 | | | | 0.111 |
| 12on | f | | 0.497 | | | 0.236 | | | 0.034 | | | | 0.961 |
| 21off | f | | 0.110 | | | 0.245 | | | -0.370 | | | | 0.590 |
| 21on | f | | 0.411 | | | 0.234 | | | -0.047 | | | | 0.869 |
| 11off | m | | 0.255 | | | 0.189 | | | -0.115 | | | | 0.626 |
| 11on | m | | 1.329 | | | 0.162 | | | 1.011 | | | | 1.647 |
| 12off | m | | -0.090 | | | 0.198 | | | -0.477 | | | | 0.297 |
| 12on | m | | 0.080 | | | 0.193 | | | -0.298 | | | | 0.457 |
| 21off | m | | -0.279 | | | 0.202 | | | -0.676 | | | | 0.117 |
| 21on | m | | 0.034 | | | 0.195 | | | -0.348 | | | | 0.416 |
| **Contrasts** | | | | | | | | | | | | | |
| **Contrast** | | **Estimate** | | | **SE** | | | **t ratio** | | | **p-value** | | |
| 11off f - 11on f | | -0.714 | | | 0.168 | | | -4.241 | | | 0.001 | | |
| 11off f - 12off f | | 1.004 | | | 0.271 | | | 3.701 | | | 0.012 | | |
| 11off f - 12on f | | 0.048 | | | 0.209 | | | 0.228 | | | 1.000 | | |
| 11off f - 21off f | | 0.435 | | | 0.222 | | | 1.955 | | | 0.724 | | |
| 11off f - 21on f | | 0.134 | | | 0.208 | | | 0.644 | | | 1.000 | | |
| 11off f - 11off m | | 0.290 | | | 0.287 | | | 1.009 | | | 0.998 | | |
| 11off f - 11on m | | -0.784 | | | 0.274 | | | -2.863 | | | 0.154 | | |
| 11off f - 12off m | | 0.635 | | | 0.294 | | | 2.164 | | | 0.576 | | |
| 11off f - 12on m | | 0.466 | | | 0.290 | | | 1.605 | | | 0.908 | | |
| 11off f - 21off m | | 0.824 | | | 0.297 | | | 2.772 | | | 0.192 | | |
| 11off f - 21on m | | 0.511 | | | 0.291 | | | 1.754 | | | 0.843 | | |
| 11on f - 12off f | | 1.718 | | | 0.252 | | | 6.805 | | | <.0001 | | |
| 11on f - 12on f | | 0.762 | | | 0.186 | | | 4.089 | | | 0.003 | | |
| 11on f - 21off f | | 1.149 | | | 0.198 | | | 5.803 | | | <.0001 | | |
| 11on f - 21on f | | 0.848 | | | 0.183 | | | 4.629 | | | 0.000 | | |
| 11on f - 11off m | | 1.004 | | | 0.270 | | | 3.714 | | | 0.011 | | |
| 11on f - 11on m | | -0.070 | | | 0.253 | | | -0.276 | | | 1.000 | | |
| 11on f - 12off m | | 1.349 | | | 0.276 | | | 4.881 | | | 0.000 | | |
| 11on f - 12on m | | 1.180 | | | 0.273 | | | 4.322 | | | 0.001 | | |
| 11on f - 21off m | | 1.538 | | | 0.280 | | | 5.495 | | | <.0001 | | |
| 11on f - 21on m | | 1.225 | | | 0.274 | | | 4.464 | | | 0.001 | | |
| **12off f - 12on f** | | **-0.956** | | | **0.279** | | | **-3.432** | | | **0.030** | | |
| 12off f - 21off f | | -0.569 | | | 0.290 | | | -1.959 | | | 0.721 | | |
| 12off f - 21on f | | -0.870 | | | 0.279 | | | -3.121 | | | 0.077 | | |
| 12off f - 11off m | | -0.714 | | | 0.342 | | | -2.091 | | | 0.630 | | |
| 12off f - 11on m | | -1.788 | | | 0.332 | | | -5.381 | | | <.0001 | | |
| 12off f - 12off m | | -0.369 | | | 0.347 | | | -1.062 | | | 0.996 | | |
| 12off f - 12on m | | -0.538 | | | 0.344 | | | -1.564 | | | 0.922 | | |
| 12off f - 21off m | | -0.180 | | | 0.351 | | | -0.513 | | | 1.000 | | |
| 12off f - 21on m | | -0.493 | | | 0.345 | | | -1.428 | | | 0.958 | | |
| 12on f - 21off f | | 0.387 | | | 0.233 | | | 1.661 | | | 0.886 | | |
| 12on f - 21on f | | 0.086 | | | 0.217 | | | 0.398 | | | 1.000 | | |
| 12on f - 11off m | | 0.242 | | | 0.293 | | | 0.825 | | | 1.000 | | |
| 12on f - 11on m | | -0.832 | | | 0.286 | | | -2.912 | | | 0.137 | | |
| 12on f - 12off m | | 0.587 | | | 0.301 | | | 1.954 | | | 0.725 | | |
| 12on f - 12on m | | 0.418 | | | 0.297 | | | 1.408 | | | 0.962 | | |
| 12on f - 21off m | | 0.776 | | | 0.305 | | | 2.544 | | | 0.313 | | |
| 12on f - 21on m | | 0.463 | | | 0.298 | | | 1.555 | | | 0.925 | | |
| **21off f - 21on f** | | **-0.301** | | | **0.232** | | | **-1.294** | | | **0.980** | | |
| 21off f - 11off m | | -0.145 | | | 0.305 | | | -0.476 | | | 1.000 | | |
| 21off f - 11on m | | -1.219 | | | 0.293 | | | -4.159 | | | 0.002 | | |
| 21off f - 12off m | | 0.200 | | | 0.311 | | | 0.643 | | | 1.000 | | |
| 21off f - 12on m | | 0.031 | | | 0.308 | | | 0.099 | | | 1.000 | | |
| 21off f - 21off m | | 0.389 | | | 0.315 | | | 1.236 | | | 0.986 | | |
| 21off f - 21on m | | 0.076 | | | 0.309 | | | 0.246 | | | 1.000 | | |
| 21on f - 11off m | | 0.156 | | | 0.294 | | | 0.529 | | | 1.000 | | |
| 21on f - 11on m | | -0.918 | | | 0.284 | | | -3.236 | | | 0.055 | | |
| 21on f - 12off m | | 0.501 | | | 0.301 | | | 1.666 | | | 0.884 | | |
| 21on f - 12on m | | 0.332 | | | 0.297 | | | 1.115 | | | 0.994 | | |
| 21on f - 21off m | | 0.690 | | | 0.305 | | | 2.263 | | | 0.503 | | |
| 21on f - 21on m | | 0.377 | | | 0.298 | | | 1.263 | | | 0.984 | | |
| **11off m - 11on m** | | **-1.074** | | | **0.123** | | | **-8.707** | | | **<.0001** | | |
| 11off m - 12off m | | 0.345 | | | 0.157 | | | 2.199 | | | 0.550 | | |
| 11off m - 12on m | | 0.176 | | | 0.150 | | | 1.171 | | | 0.991 | | |
| 11off m - 21off m | | 0.534 | | | 0.165 | | | 3.229 | | | 0.056 | | |
| 11off m - 21on m | | 0.221 | | | 0.152 | | | 1.453 | | | 0.953 | | |
| 11on m - 12off m | | 1.419 | | | 0.136 | | | 10.435 | | | <.0001 | | |
| 11on m - 12on m | | 1.250 | | | 0.129 | | | 9.680 | | | <.0001 | | |
| 11on m - 21off m | | 1.608 | | | 0.143 | | | 11.224 | | | <.0001 | | |
| 11on m - 21on m | | 1.295 | | | 0.132 | | | 9.800 | | | <.0001 | | |
| **12off m - 12on m** | | **-0.170** | | | **0.163** | | | **-1.041** | | | **0.997** | | |
| 12off m - 21off m | | 0.189 | | | 0.176 | | | 1.070 | | | 0.996 | | |
| 12off m - 21on m | | -0.124 | | | 0.165 | | | -0.753 | | | 1.000 | | |
| 12on m - 21off m | | 0.359 | | | 0.171 | | | 2.100 | | | 0.623 | | |
| 12on m - 21on m | | 0.045 | | | 0.158 | | | 0.286 | | | 1.000 | | |
| **21off m - 21on m** | | **-0.313** | | | **0.173** | | | **-1.813** | | | **0.811** | | |

**Table S4 -** Summary and details of the GLMM and pairwise post-hoc test searching for the effect of r_k_ interval type (on-integer and off-integer for each ratio for 1:1, 1:2 and 2:1 category) and sex on the observation count (*i.e.* number of observed r_k_) in **ADULTS**.

**a -** Summary of the full model - Influence of the fixed factors (r_k_ interval: 11 on - 1off - 12on - 12off - 21on - 21off; Sex: Female - Male) on the count of the observations. The identification code of the contribution is the random factor (singer ID)

**b -** Results for the pairwise post-hoc test on the interaction r_k_ interval * sex

| **a**  **Generalized Linear Model**  Family: beta  Count (r_k_ obs) ~ r_k_ interval * sex + offset + (singer ID)  Full vs Null (Chisq=3943.574; df=11; p<.0001) | | | | | | | | | | | | | |
| --- | --- | --- | --- | --- | --- | --- | --- | --- | --- | --- | --- | --- | --- |
| **Variable** | | | | **Estimate** | | | **SE** | | | **z value** | | **p-value (z)** | |
| (Intercept) ^b,c^ | | | | 0.731 | | | 0.114 | | | ^a^ | | ^a^ | |
| r_k_ interval (11on) ^b,c^ | | | | 0.922 | | | 0.029 | | | 32.270 | | <.0001 | |
| r_k_ interval (12off) ^b,c^ | | | | -0.105 | | | 0.036 | | | -2.900 | | 0.004 | |
| r_k_ interval (12on) ^b,c^ | | | | 0.213 | | | 0.033 | | | 6.370 | | <.0001 | |
| r_k_ interval (21off) ^b,c^ | | | | -0.129 | | | 0.036 | | | -3.570 | | <.001 | |
| r_k_ interval (21on) ^b,c^ | | | | 0.062 | | | 0.034 | | | 1.800 | | 0.071 | |
| sex (m) ^b,c^ | | | | -0.550 | | | 0.154 | | | -3.580 | | <.001 | |
| r_k_ interval (11on): sex (m) ^b,c^ | | | | 0.049 | | | 0.047 | | | 1.040 | | 0.300 | |
| r_k_ interval (12off): sex (m) ^b,c^ | | | | -0.168 | | | 0.061 | | | -2.740 | | 0.006 | |
| r_k_ interval (12on): sex (m) ^b,c^ | | | | 0.111 | | | 0.055 | | | 2.020 | | 0.043 | |
| r_k_ interval (21off): sex (m) ^b,c^ | | | | -0.142 | | | 0.061 | | | -2.330 | | 0.020 | |
| r_k_ interval (21on): sex (m) ^b,c^ | | | | 0.157 | | | 0.056 | | | 2.790 | | 0.005 | |
| ^a^ Not shown as not having a meaningful interpretation  ^b^ Estimate ± SE refer to the difference of the response between the reported level of this categorical predictor and the reference category of the same predictor  ^c^ Reference categories: “r_k_ interval (11off)”, “sex (f)” | | | | | | | | | | | | | |
| **b**  ***Post-hoc* comparisons** | | | | | | | | | | | | | |
| **Emmeans** | | | | | | | | | | | | | |
| **r_k_ interval** | **sex** | | **emmean** | | | **SE** | | | **lower.CL** | | | | **upper.CL** |
| 11off | f | | 0.803 | | | 0.114 | | | 0.579 | | | | 1.027 |
| 11on | f | | 1.725 | | | 0.112 | | | 1.505 | | | | 1.946 |
| 12off | f | | 0.699 | | | 0.115 | | | 0.474 | | | | 0.923 |
| 12on | f | | 1.016 | | | 0.114 | | | 0.792 | | | | 1.239 |
| 21off | f | | 0.674 | | | 0.115 | | | 0.450 | | | | 0.899 |
| 21on | f | | 0.865 | | | 0.114 | | | 0.641 | | | | 1.089 |
| 11off | m | | 0.253 | | | 0.103 | | | 0.051 | | | | 0.455 |
| 11on | m | | 1.224 | | | 0.099 | | | 1.030 | | | | 1.418 |
| 12off | m | | -0.019 | | | 0.104 | | | -0.224 | | | | 0.185 |
| 12on | m | | 0.577 | | | 0.102 | | | 0.377 | | | | 0.776 |
| 21off | m | | -0.018 | | | 0.104 | | | -0.222 | | | | 0.187 |
| 21on | m | | 0.472 | | | 0.102 | | | 0.272 | | | | 0.672 |
| **Contrasts** | | | | | | | | | | | | | |
| **Contrast** | | **Estimate** | | | **SE** | | | **t ratio** | | | **p-value** | | |
| **11off f - 11on f** | | **-0.922** | | | **0.029** | | | **-32.272** | | | **<.0001** | | |
| 11off f - 12off f | | 0.105 | | | 0.036 | | | 2.900 | | | 0.141 | | |
| 11off f - 12on f | | -0.213 | | | 0.033 | | | -6.375 | | | <.0001 | | |
| 11off f - 21off f | | 0.129 | | | 0.036 | | | 3.575 | | | 0.018 | | |
| 11off f - 21on f | | -0.062 | | | 0.035 | | | -1.804 | | | 0.816 | | |
| 11off f - 11off m | | 0.550 | | | 0.154 | | | 3.580 | | | 0.018 | | |
| 11off f - 11on m | | -0.421 | | | 0.151 | | | -2.784 | | | 0.187 | | |
| 11off f - 12off m | | 0.822 | | | 0.155 | | | 5.320 | | | <.0001 | | |
| 11off f - 12on m | | 0.226 | | | 0.153 | | | 1.481 | | | 0.946 | | |
| 11off f - 21off m | | 0.821 | | | 0.155 | | | 5.310 | | | <.0001 | | |
| 11off f - 21on m | | 0.331 | | | 0.153 | | | 2.163 | | | 0.577 | | |
| 11on f - 12off f | | 1.027 | | | 0.030 | | | 34.051 | | | <.0001 | | |
| 11on f - 12on f | | 0.710 | | | 0.027 | | | 26.533 | | | <.0001 | | |
| 11on f - 21off f | | 1.051 | | | 0.030 | | | 35.043 | | | <.0001 | | |
| 11on f - 21on f | | 0.860 | | | 0.028 | | | 30.544 | | | <.0001 | | |
| 11on f - 11off m | | 1.472 | | | 0.153 | | | 9.657 | | | <.0001 | | |
| 11on f - 11on m | | 0.501 | | | 0.150 | | | 3.343 | | | 0.040 | | |
| 11on f - 12off m | | 1.745 | | | 0.153 | | | 11.378 | | | <.0001 | | |
| 11on f - 12on m | | 1.149 | | | 0.152 | | | 7.576 | | | <.0001 | | |
| 11on f - 21off m | | 1.743 | | | 0.153 | | | 11.369 | | | <.0001 | | |
| 11on f - 21on m | | 1.253 | | | 0.152 | | | 8.255 | | | <.0001 | | |
| **12off f - 12on f** | | **-0.317** | | | **0.035** | | | **-9.161** | | | **<.0001** | | |
| 12off f - 21off f | | 0.024 | | | 0.037 | | | 0.648 | | | 1.000 | | |
| 12off f - 21on f | | -0.167 | | | 0.036 | | | -4.670 | | | <.001 | | |
| 12off f - 11off m | | 0.446 | | | 0.154 | | | 2.895 | | | 0.143 | | |
| 12off f - 11on m | | -0.526 | | | 0.152 | | | -3.468 | | | 0.026 | | |
| 12off f - 12off m | | 0.718 | | | 0.155 | | | 4.636 | | | <.001 | | |
| 12off f - 12on m | | 0.122 | | | 0.153 | | | 0.796 | | | 1.000 | | |
| 12off f - 21off m | | 0.716 | | | 0.155 | | | 4.626 | | | <.001 | | |
| 12off f - 21on m | | 0.227 | | | 0.153 | | | 1.478 | | | 0.947 | | |
| 12on f - 21off f | | 0.341 | | | 0.035 | | | 9.879 | | | <.0001 | | |
| 12on f - 21on f | | 0.150 | | | 0.033 | | | 4.563 | | | <.001 | | |
| 12on f - 11off m | | 0.763 | | | 0.153 | | | 4.973 | | | <.0001 | | |
| 12on f - 11on m | | -0.209 | | | 0.151 | | | -1.382 | | | 0.967 | | |
| 12on f - 12off m | | 1.035 | | | 0.154 | | | 6.709 | | | <.0001 | | |
| 12on f - 12on m | | 0.439 | | | 0.153 | | | 2.878 | | | 0.149 | | |
| 12on f - 21off m | | 1.033 | | | 0.154 | | | 6.699 | | | <.0001 | | |
| 12on f - 21on m | | 0.544 | | | 0.153 | | | 3.559 | | | 0.019 | | |
| **21off f - 21on f** | | **-0.191** | | | **0.036** | | | **-5.355** | | | **<.0001** | | |
| 21off f - 11off m | | 0.422 | | | 0.154 | | | 2.739 | | | 0.207 | | |
| 21off f - 11on m | | -0.550 | | | 0.152 | | | -3.628 | | | 0.015 | | |
| 21off f - 12off m | | 0.694 | | | 0.155 | | | 4.481 | | | 0.001 | | |
| 21off f - 12on m | | 0.098 | | | 0.153 | | | 0.639 | | | 1.000 | | |
| 21off f - 21off m | | 0.692 | | | 0.155 | | | 4.471 | | | 0.001 | | |
| 21off f - 21on m | | 0.203 | | | 0.153 | | | 1.321 | | | 0.977 | | |
| 21on f - 11off m | | 0.612 | | | 0.154 | | | 3.987 | | | 0.004 | | |
| 21on f - 11on m | | -0.359 | | | 0.151 | | | -2.375 | | | 0.423 | | |
| 21on f - 12off m | | 0.885 | | | 0.155 | | | 5.726 | | | <.0001 | | |
| 21on f - 12on m | | 0.289 | | | 0.153 | | | 1.889 | | | 0.766 | | |
| 21on f - 21off m | | 0.883 | | | 0.154 | | | 5.716 | | | <.0001 | | |
| 21on f - 21on m | | 0.393 | | | 0.153 | | | 2.571 | | | 0.297 | | |
| 11off m - 11on m | | -0.971 | | | 0.038 | | | -25.388 | | | <.0001 | | |
| 11off m - 12off m | | 0.272 | | | 0.050 | | | 5.503 | | | <.0001 | | |
| **11off m - 12on m** | | **-0.324** | | | **0.044** | | | **-7.421** | | | **<.0001** | | |
| 11off m - 21off m | | 0.270 | | | 0.049 | | | 5.488 | | | <.0001 | | |
| 11off m - 21on m | | -0.219 | | | 0.044 | | | -4.941 | | | <.0001 | | |
| 11on m - 12off m | | 1.243 | | | 0.042 | | | 29.946 | | | <.0001 | | |
| 11on m - 12on m | | 0.648 | | | 0.034 | | | 18.843 | | | <.0001 | | |
| 11on m - 21off m | | 1.242 | | | 0.041 | | | 30.128 | | | <.0001 | | |
| 11on m - 21on m | | 0.752 | | | 0.035 | | | 21.356 | | | <.0001 | | |
| **12off m - 12on m** | | **-0.596** | | | **0.047** | | | **-12.770** | | | **<.0001** | | |
| 12off m - 21off m | | -0.002 | | | 0.052 | | | -0.034 | | | 1.000 | | |
| 12off m - 21on m | | -0.491 | | | 0.047 | | | -10.382 | | | <.0001 | | |
| 12on m - 21off m | | 0.594 | | | 0.046 | | | 12.794 | | | <.0001 | | |
| 12on m - 21on m | | 0.105 | | | 0.041 | | | 2.547 | | | 0.311 | | |
| **21off m - 21on m** | | **-0.490** | | | **0.047** | | | **-10.392** | | | **<.0001** | | |

**Table S5** - Summary and details of the GLMM and pairwise post-hoc test searching for the effect of sex and age on the **rhythmic stability** around 1:1 small-integer.

**a -** Summary of the full model - Influence of the fixed factors (Sex: Female - Male; Age: 0-2, 3-4, Adults) on the rhythmic stability (quantified as the ratio between the number of rk falling inside the on-isochrony boundaries divided per the number of rk falling inside the on-isochrony boundaries plus the number of rk falling inside the off-isochrony boundaries). The code of the contribution is the random factor (contribution ID)

**b -** Results for the pairwise post-hoc test on the interaction between age*sex

| **a**  **Generalized Linear Model**  Family: beta  Rhythmic stability ~ age * sex + (contribution ID)  Full vs Null (Chisq=21.519; df=5; p<0.001) | | | | | | | | | | | | | |
| --- | --- | --- | --- | --- | --- | --- | --- | --- | --- | --- | --- | --- | --- |
| **Variable** | | **Estimate** | | | | **SE** | | | **z value** | | | **p-value (z)** | |
| (Intercept) ^b,c^ | | 1.074 | | | | 0.218 | | | ^a^ | | | ^a^ | |
| Age (3-4) ^b.c^ | | -0.031 | | | | 0.352 | | | -0.089 | | | 0.929 | |
| Age (adult) | | -0.059 | | | | 0.225 | | | -0.262 | | | 0.793 | |
| Sex (m) ^b.c^ | | 0.770 | | | | 0.397 | | | 1.941 | | | 0.052 | |
| Age (3-4) : sex (m) ^b.c^ | | -0.317 | | | | 0.510 | | | -0.621 | | | 0.535 | |
| Age (adults) : sex (m) ^b.c^ | | -0.491 | | | | 0.404 | | | -1.215 | | | 0.225 | |
| ^a^ Not shown as not having a meaningful interpretation  ^b^ Estimate ± SE refer to the difference of the response between the reported level of this categorical predictor and the reference category of the same predictor  ^c^ Reference categories: “age (0-2)”, “sex (f)” | | | | | | | | | | | | | |
| **b**  ***Post-hoc* comparisons** | | | | | | | | | | | | | |
| **Emmeans** | | | | | | | | | | | | | |
| **Age** | **sex** | **emmean** | | **SE** | | | **lower.CL** | | | **upper.CL** | | |  |
| 0-2 | f | 1.070 | | 0.218 | | | 0.646 | | | 1.500 | | |  |
| 3-4 | f | 1.040 | | 0.278 | | | 0.499 | | | 1.590 | | |  |
| Adult | f | 1.020 | | 0.060 | | | 0.898 | | | 1.130 | | |  |
| 0-2 | m | 1.840 | | 0.333 | | | 1.191 | | | 2.500 | | |  |
| 3-4 | m | 1.500 | | 0.163 | | | 1.178 | | | 1.810 | | |  |
| Adult | m | 1.290 | | 0.056 | | | 1.184 | | | 1.400 | | |  |
| **Contrasts** | | | | | | | | | | | | |  |
| **Contrast** | | | **Estimate** | | **SE** | | | **t ratio** | | | **p-value** | |  |
| 0-2 f - 3-4 f | | | 0.031 | | 0.352 | | | 0.089 | | | 1.000 | |  |
| 0-2 f - adult f | | | 0.059 | | 0.225 | | | 0.262 | | | 1.000 | |  |
| 0-2 f - 0-2 m | | | -0.770 | | 0.397 | | | -1.941 | | | 0.377 | |  |
| 0-2 f - 3-4 m | | | -0.422 | | 0.271 | | | -1.560 | | | 0.625 | |  |
| 0-2 f - adult m | | | -0.220 | | 0.224 | | | -0.984 | | | 0.923 | |  |
| 3-4 f - adult f | | | 0.028 | | 0.283 | | | 0.098 | | | 1.000 | |  |
| 3-4 f - 0-2 m | | | -0.801 | | 0.432 | | | -1.853 | | | 0.432 | |  |
| 3-4 f - 3-4 m | | | -0.453 | | 0.321 | | | -1.414 | | | 0.718 | |  |
| 3-4 f - adult m | | | -0.251 | | 0.282 | | | -0.891 | | | 0.949 | |  |
| adult f - 0-2 m | | | -0.829 | | 0.336 | | | -2.463 | | | 0.135 | |  |
| adult f - 3-4 m | | | -0.481 | | 0.171 | | | -2.818 | | | 0.055 | |  |
| adult f - adult m | | | -0.279 | | 0.077 | | | -3.603 | | | 0.004 | |  |
| 0-2 m - 3-4 m | | | 0.348 | | 0.368 | | | 0.944 | | | 0.935 | |  |
| 0-2 m - adult m | | | 0.550 | | 0.336 | | | 1.637 | | | 0.574 | |  |
| 3-4 m - adult m | | | 0.202 | | 0.170 | | | 1.191 | | | 0.841 | |  |
